# New Close Binary Central Stars of Planetary Nebulae from Gaia DR3 Epoch Photometry

**DOI:** 10.3847/2515-5172/ac8e6c

**Published:** 2022-09-05

**Authors:** Nicholas Chornay, Nicholas A. Walton

**Affiliations:** 1 Institute of Astronomy, University of Cambridge, Madingley Road, Cambridge CB3 0HA, UK; njc89@cam.ac.uk

## Abstract

Close binary interactions perform a key role in the formation and shaping of planetary nebulae (PNe). However only a small fraction of Galactic PNe are known to host close binary systems. Many such systems are detectable through photometric variability. We searched recently published epoch photometry data from Gaia DR3 for planetary nebula central stars with periodic photometric variability indicative of binarity, uncovering four previously unknown close binaries.

## 1. Introduction

A significant fraction of planetary nebulae (PNe) have central stars (CSPNe) that evolved in close binary systems (Jones & Boffin 2017). However only around a hundred such systems are known out of a few thousand PNe (though estimates of photometrically detectable fraction are on the order of 20%: Miszalski et al. 2009; Jacoby et al. 2021). Expanding the sample further will improve statistical significance and allow stronger conclusions to be drawn about the properties of these objects.

Many close binary CSPNe have been identified from their photometric variability (due to irradiation, ellipsoidal modulation, and eclipses; Boffin & Jones 2019), both as a result of dedicated monitoring (e.g., Corradi et al. 2011) and in data from larger surveys (e.g., Miszalski et al. 2009). More recently new binaries have been found using space-based photometry from missions such as Kepler/K2 (Jacoby et al. 2021) and Gaia (Chornay et al. 2021). In this work we report on new discoveries from the epoch (time series) photometry released as part of Gaia Data Release 3 (DR3; Gaia Collaboration et al. 2022).

## 2. Methods

Epoch photometric data is published in Gaia DR3 for objects classified as variable in its data processing (Eyer et al. 2022). We cross-matched the CSPN catalog of Chornay & Walton (2021) (including unpublished low-confidence matches for completeness) with the variable sources in Gaia DR3 and retrieved epoch photometry for the 126 sources that resulted from the cross-match. As was done in Chornay et al. (2021), we performed a Lomb-Scargle analysis (using the implementation of VanderPlas & Ivezić 2015) on the *G*-band light curves in order to search for periodic variability on timescales between hours and weeks. The resulting periodograms and folded light curves were inspected for indications of close binarity.

## 3. Results

We identified four CSPNe whose Gaia DR3 light curves show strong signatures of close binarity, and which were not previously known to be binaries.^1^ 
^1^
Based on the list compiled at https://www.drdjones.net/bcspn/, as of 2021 August 24th. There are other CSPNe that exhibit periodic variability in the Gaia data, but for which the origin is less clear, due to high scatter (e.g. Kn 51), low amplitude (e.g. NGC 6891), unclear association of the source with the PN (e.g. BRAN 229), or sparse sampling. These are left for future work.

**Figure 1.**
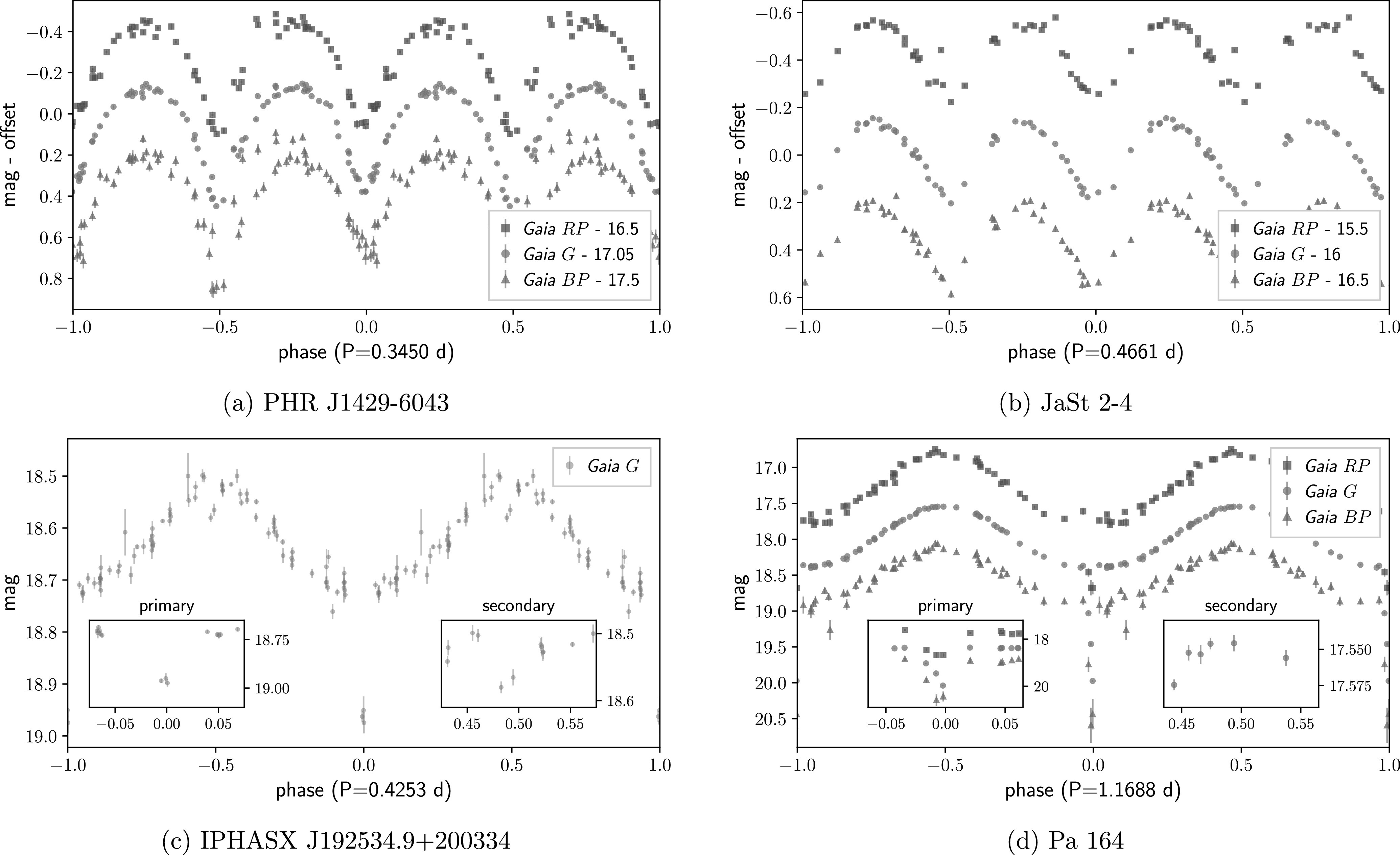
Folded Gaia light curves for the four close binary systems identified in this work.

*PHR J1429–6043:*this is a faint, extended “possible” PN (PN G314.6–00.1) from the Macquarie/AAO/Strasbourg H*α* PNe catalog (MASH; Parker et al. 2006). The identity of the CSPN in Gaia has been uncertain, with Chornay & Walton (2021) not publishing a match. Gaia DR3 reveals that the closest source to the center of the nebula (Gaia DR3 5878618052702285056, *α* = 14^h^29^m^53.ˢ08  *δ* = −60°43″56.′25) is indeed the CSPN, an ellipsoidally modulated system with a ∼8.3 hr orbital period (Figure 1(a); magnitudes shown in the correct relative order but offset for clarity). The Gaia light curve shows small differences in the depths of the minima, typical of a system in which the two stars have different masses (Boffin & Jones 2019).

*JaSt 2–4:*this is a faint, slightly elliptical ring PN (PN G001.0+01.4) discovered by Jacoby & Van de Steene (2004). The Gaia photometric data of its CSPN (Gaia DR3 4060890513224820352, *α* = 17^h^42^m^28.ˢ06  *δ* = −27°13″31.′79) shows ellipsoidal modulation effects corresponding to an orbital period of ∼11.2 hr (Figure 1(b); magnitudes are offset).

*IPHASX J192534.9+200334:*this is an irregularly shaped “likely” PN (PN G054.5+01.8) discovered by Sabin et al. (2014). Its CSPN is also missing from Chornay & Walton (2021), but the Gaia DR3 data for the closest source to the center of the nebula (Gaia DR3 4515887189511585792, $\alpha ={19}^{{\rm{h}}}{25}^{{\rm{m}}}34\buildrel{\rm{s}}\over{.} 90\ \delta =+20^\circ 03^{\prime\prime} 34\buildrel{\,\prime}\over{.} 74$) shows that it is indeed the CSPN, a doubly eclipsing binary system with a ∼10.2 hr period and sinusoidal brightness variability due to irradiation of the cooler companion (Figure 1(c)).

*Pa 164:*this is a “likely” PN (PN G061.5–02.6) from Kronberger et al. (2017). The Gaia light curve of its CSPN (Gaia DR3 1834171384397003264, $\alpha ={19}^{{\rm{h}}}{57}^{{\rm{m}}}23\buildrel{\rm{s}}\over{.} 23\ \delta =+23^\circ 52^{\prime\prime} 48\buildrel{\,\prime}\over{.} 27$) shows it to be an eclipsing irradiated binary with a period of ∼28 hr (Figure 1(d)). A secondary eclipse is not evident in the Gaia data. It was published as a candidate variable by Chornay et al. (2021).

## 4. Conclusion

We have found four new close binary CSPNe using the Gaia DR3 epoch photometry data. Two of the sources had not been previously identified as CSPNe, because of the extended natures of their nebulae and their lack of obvious blue colors expected for CSPNe. This highlights a difficulty in CSPN identification especially applicable to close binaries, where a significant contribution to the flux can come from the cooler companion star.

We note that about a quarter of the previously known close binary CSPN population (26 objects) have matching sources classified as variable in Gaia DR3. Our analysis recovers the correct period (or half of it, in the case of ellipsoidal modulation) for 21 of these objects. For three of these objects the shape of folded light curve is not obviously due to binarity, despite the period being correct. Thus it is very likely that there are more close binaries waiting to be discovered in the Gaia photometry, though these will benefit from ground-based confirmation.

Gaia DR3 is based on 34 months of data. The duration of the data collection will be nearly doubled in the next data release, with the full mission duration expected to be over ten years.^2^ 
^2^

https://www.cosmos.esa.int/web/gaia/release
 Future releases will thus benefit from a longer baseline and many more data points (Eyer et al. 2022), which will continue to make Gaia a valuable tool for probing CSPN variability. This is particularly true for systems where bright nebulae or nearby stars complicate ground-based photometric monitoring.
